# Characterization of Information-Based Learning Benefits with Submovement Dynamics and Muscular Rhythmicity

**DOI:** 10.1371/journal.pone.0082920

**Published:** 2013-12-18

**Authors:** Ing-Shiou Hwang, Chien-Ting Huang, Jeng-Feng Yang, Mei-Chun Guo

**Affiliations:** 1 Department of Physical Therapy, College of Medicine, National Cheng Kung University, Tainan, Taiwan; 2 Institute of Allied Health Sciences, College of Medicine, National Cheng Kung University, Tainan, Taiwan; SUNY Downstate MC, United States of America

## Abstract

For skill advancement, motor variability must be optimized based on target information during practice sessions. This study investigated structural changes in kinematic variability by characterizing submovement dynamics and muscular oscillations after practice with visuomotor tracking under different target conditions. Thirty-six participants were randomly assigned to one of three groups (simple, complex, and random). Each group practiced tracking visual targets with trajectories of varying complexity. The velocity trajectory of tracking was decomposed into 1) a primary contraction spectrally identical to the target rate and 2) an intermittent submovement profile. The learning benefits and submovement dynamics were conditional upon experimental manipulation of the target information. Only the simple and complex groups improved their skills with practice. The size of the submovements was most greatly reduced by practice with the least target information (simple > complex > random). Submovement complexity changed in parallel with learning benefits, with the most remarkable increase in practice under a moderate amount of target information (complex > simple > random). In the simple and complex protocols, skill improvements were associated with a significant decline in alpha (8–12 Hz) muscular oscillation but a potentiation of gamma (35–50 Hz) muscular oscillation. However, the random group showed no significant change in tracking skill or submovement dynamics, except that alpha muscular oscillation was reduced. In conclusion, submovement and gamma muscular oscillation are biological markers of learning benefits. Effective learning with an appropriate amount of target information reduces the size of submovements. In accordance with the challenge point hypothesis, changes in submovement complexity in response to target information had an inverted-U function, pertaining to an abundant trajectory-tuning strategy with target exactness.

## Introduction

Motor learning produces relatively permanent increases in motor success and performance consistency. Although variations in motion could degrade task performance, motor variability is not completely negative, as it can be integral to the exploration of drive actions [1], [2]. Some motor variability is necessary for shaping undesired movement patterns [3]. Research has shown that practice could reduce the size of motor variability [4], [5] and alter the structure of motor variability in terms of reductions in noise level and error tolerance [6], [7], [8]. For one-dimensional kinematic data, the structure of motor variability is typically indexed with entropy measures, which characterize the degree of predictability of kinematic fluctuations over a data stream. Entropy changes in movement fluctuations with effective practice are subject to engagement of error corrections with sensory feedback [9], [10] and/or adaptability to environmental perturbations [11].

One of the major sources of motor variability is submovements, which manifest as numerous pulse elements in the movement trajectory [12], [13]. Submovements are generated because the central nervous system plans and approximates the desired movement pattern with sampled processes against feedback delays [14], [15], [16]. Namely, central scaling of submovements is a part of an additive accuracy control during visuomotor tracking [13]. Structure of submovements in the movement trajectory changes with manipulation of visual feedback [17], [18]. Increase in target rate [17], enhanced intermittent frequency of visual information [18], or removal of online visual feedback [15], [19] during visuomotor tracking leads to a reduction in the signs of submovements, due to interruption of actor's corrective actions with feedback processes. Although movement reorganization due to motor learning has been analyzed at a number of levels, little attention has been paid to changes in the submovement dynamics [20] underlying the strategic rebalancing of the feedback and feed-forward processes after learning [18], [21].

The amount of information in a learning paradigm determines task difficulty and learning benefits. According to the challenge point framework [22], learning benefits are optimized with the amount of skill-dependent task information. Learning alters the transmission of information from inputs to outputs and dimensional changes in the state space of observed behaviors [6], [7]. A prevailing view is that learning reduces dynamic degrees of freedom (dimension); however, a dimensional increase is possible for learning a challenging motor task, known as motor abundance [23]. The present study aims to map the learning benefits of single-joint visuomotor tracking onto submovement characteristics under the condition of varied target information. Our main hypotheses are that 1) the size and the complexity of submovements will be differently modulated with the amount of target information in a learning paradigm, and 2) learning benefits under different target constraints will be represented by practice-related changes in submovement dynamics. This study also links the practice-related submovement scaling to muscular oscillatory activities normally present in phasic movements [24], [25], [26]. Theoretically, characterizing submovement dynamics and muscular oscillation will lend insight into the fine-tuning and internal coding of movement trajectories associated with information-based learning.

## Materials and Methods

### Ethics Statement

The experiment was approved by the local human experiment and ethics committee (National Cheng Kung University Hospital Institutional Review Board, NCKUH IRB), and written informed consent was obtained from all participants before the experiment, conforming to the Declaration of Helsinki.

### Subjects

A total of thirty-six right-handed young adults (19 female and 17 male, age: 20–29 years) without any history of neuromuscular disorders participated in this study. Handedness was tested according to the Oldfield questionnaire.

### Behavioral task

**Figure 1:**
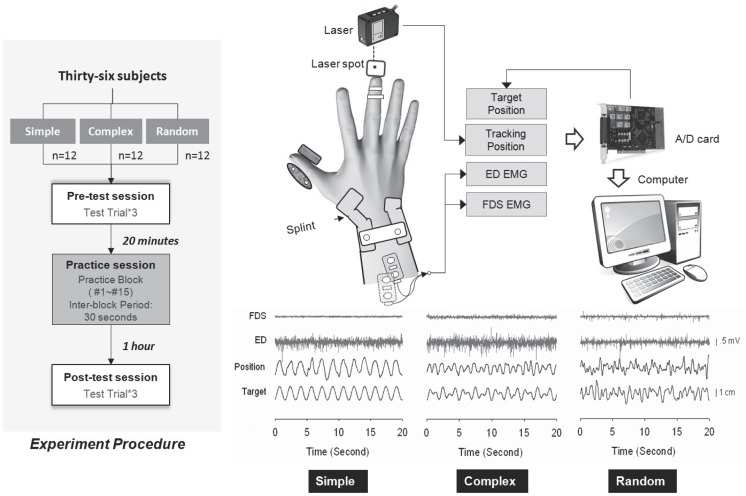
Experiment procedure, system setup, and data recording.

The subjects were randomly assigned to three experimental groups of twelve subjects, trained in one of three tracking protocols (simple, complex, and random). The subjects in the simple protocol learned to track a 0.5 Hz sinusoidal target within a range of 3 cm (±1.5 cm), and the subjects in the complex protocol learned to track a combined wave of 0.25 Hz and 0.75 Hz with 1.5 cm (±0.75 cm) of amplitude for each sinusoidal component. The subjects in the random protocol learned to pursue a quasi-random target spectrally ranging from 0 to 1 Hz. By conditioning of white noise with a 1 Hz low-pass filter, the peak-to-peak amplitude of the quasi-random target was empirically adjusted to 3 cm. The means of target rate were theoretically identical for all tracking protocols. A target rate below 1 Hz was chosen for the simple, complex, and random protocols to stimulate the use of feedback processes in the control of the tracking maneuvers [18], [27]. The target complexity (or information) of the practice protocol increased with the spectral components in a target signal (random > complex > simple). There were 3 pre-test trials (1-min rest between trials), 15 training trials (1-min rest between training trials), and 3 post-test trials (1-min rest between trials) of 20 seconds per trial for each group (Fig. 1, left). The training session began 20 minutes after the end of the last pre-test trial, and the post-test assessment was performed 1 hour after the last training trial was completed. During the experiment, a subject sat on a chair with his/her wrist and forearm of the practice (right) limb fixed within a thermoplastic splint on the table. No finger movements were constrained by the thermoplastic splint, and the fingers remained fully extended in parallel to the ground during the experiment. By carefully controlling the flexion-extension of the right metacarpophalangeal (MCP) joint, the subjects in these trials were required to couple the positional trace of the fingertip of the extended middle finger to the designated target movement with visual feedback.

### Recordings

A displacement-transducing laser (ZX-LD100, Omron, Kyoto, Japan) was placed 4 cm above the fingertip (the midpoint of its recording range)(Fig. 1, right). The laser device could measure the distance between itself and the finger tip, such that the positional trajectory of the middle finger in reference to the midpoint of the recording range was registered during manual tracking. Target signals were generated by an analog-digital card with programmable analog outputs. Target signals and displacement of the middle finger were displayed on a computer screen as a visual guide for the manual tracking. Muscle activities of the extensor digitorum (ED) and the flexor digitorum superficialis (FDS) were measured with a pair of bipolar surface electrode units (1.1 cm in diameter, gain = 365, CMRR = 102 dB, Imoed Inc., Salt Lake City, UT, USA). The electrode on the ED was placed over the muscle about three quarters of the distance between the elbow and the wrist from the elbow. The muscle activity of the FDS was recorded by placing the electrode on an oblique angle approximately 4 cm above the wrist on the palpable muscle mass. All physiological measures, including target movement, finger trajectory, and EMG signals, were digitized at 1 kHz via a 16-bit A/D converter (NI USB-6218, National Instruments, Austin, TX, USA) controlled by a computer program constructed on a LabView platform (LabView v.8.0, National Instruments, Austin, TX, USA).

### Analysis

Target signal and finger position traces were conditioned with a low-pass filter (cut-off frequency: 6 Hz)[13], [17]. EMG recordings from the ED and FDS muscles were band-pass filtered (pass-band: 1∼400 Hz) to preclude a potential linear trend of a biased current and sinusoidal movement artifacts. All conditioned physiological signals in the first and last 1 second were excluded from subsequent analyses.

#### Tracking Performance

Tracking performance was assessed with tracking congruency, defined as the cross correlation maximum (CM) between the target trajectory and the positional trace of the middle finger. A higher CM represented better tracking congruency and a higher degree of trajectory similarity between the target and the manual output. In terms of CM, tracking congruency was determined from all the experiment trials in the pre-test, practice, and post-test sessions. CM of the three trials in the pre-test and post-test sessions was averaged to assess baseline and post-practice performances. The learning benefit after practice was defined as a standardized change in the mean tracking congruency between the pre-test and post-test sessions (Δ tracking congruency), or (CM_post-test_ - CM_pre-test_)/CM_pre-test_.

#### Submovement

**Figure 2:**
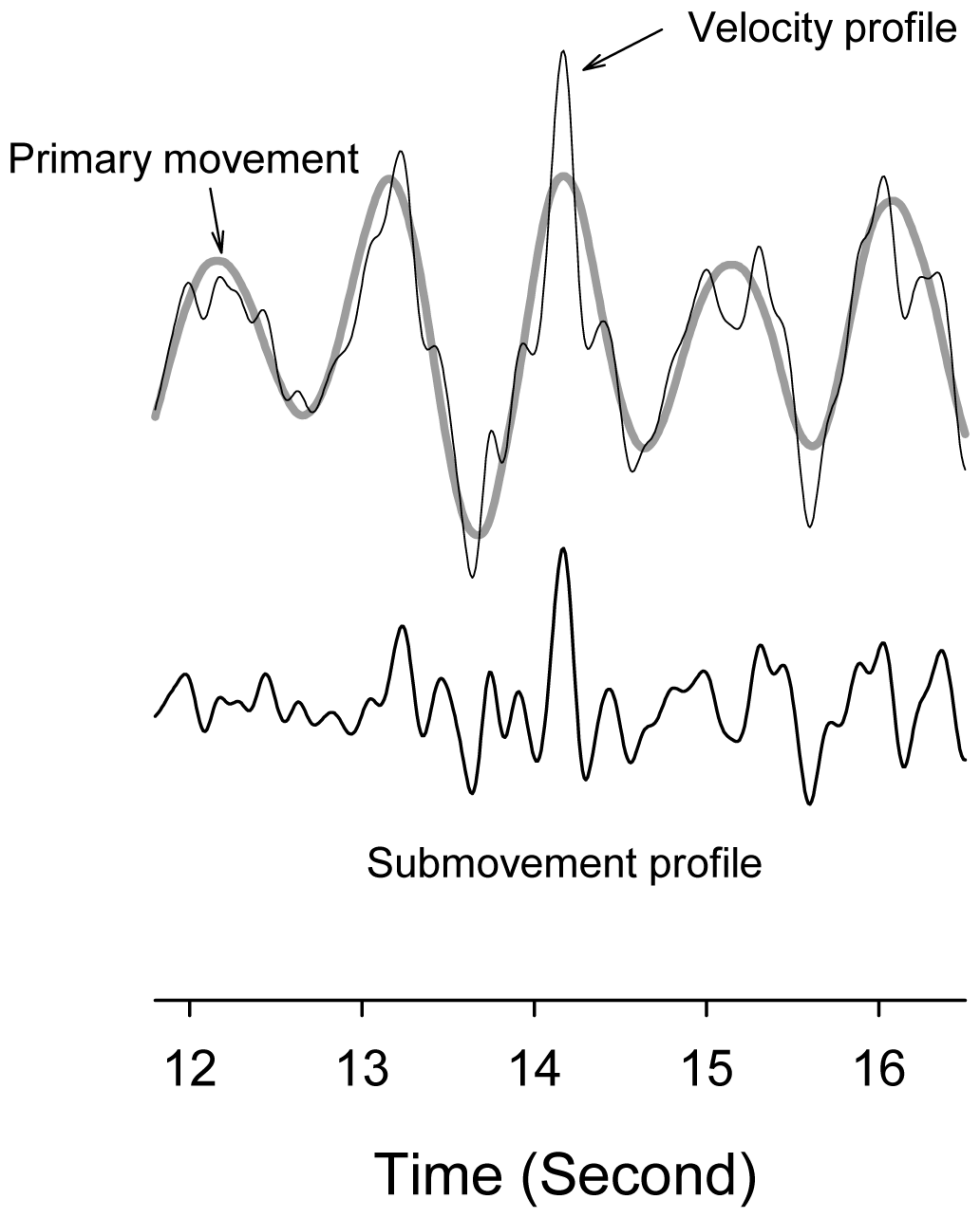
Schematic illustration of acquisition of primary movement and submovement profile from velocity profile during tracking.

Displacement data of the middle finger were down-sampled to 100 Hz in submovement analysis. The displacement data were differentiated to obtain a velocity profile. Then the velocity profile was further decomposed into two time series, the primary movement and the submovement profile (Fig. 2)[13], [17]. Spectrally identical to the moving target, the amplitude of the hypothetical primary movement approximated the velocity profile of a target signal in amplitude. The primary movement was the a priori standard of intended movement with a deterministic nature, specifically synchronized with the moving target. On the other hand, the submovement profile was of a stochastic nature, pertaining to the additive mechanisms of error correction to remedy tracking deviations [13], [17]. For the simple protocol, the submovement profile was obtained by conditioning the velocity profile with a zero-phasing notch filter that passes all frequencies except for the target rate at 0.5 Hz. The transfer function of the notch filter was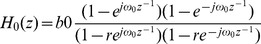, *r* = .9975, *ω_0_* = *π*/360. Subtracting the submovement profile from the velocity profile gave the primary movement, a 0.5 Hz sinusoidal wave with its amplitude approximating the velocity profile of the target signal. For the complex protocol, the submovement profile was obtained by conditioning the velocity profile with a zero-phasing notch filter to exclude frequencies of the target rate at 0.25 Hz and 0.75 Hz. The transfer function of the notch filter was 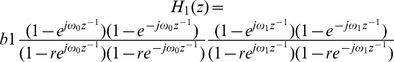, *r* = .9975, *ω_0_* = *π*/720, *ω_1_* = 3*π*/720. The primary movement was a combined sinusoidal wave of 0.25 Hz and 0.75 Hz with its amplitude approximating the velocity profile of the target signal. For the random protocol, the velocity profile of manual tracking was conditioned with a zero-phase 4th order Butterworth low-pass filter (cut-off frequency: 1 Hz). The spectral components of the velocity profile that were greater than 1 Hz (1–6 Hz) were considered as submovements, since they did not spectrally couple the target movement in the random condition.

Root mean square (RMS) was applied to obtain a time-averaged amplitude of a submovement profile and a primary movement. The relative submovement amplitude was defined as the ratio of the submovement RMS to the RMS value of the velocity profile of manual tracking. R_PM/S_ was the amplitude ratio of the primary movement relative to the submovement profile. Multi-scale entropy (MSE) analysis was a complexity measure used to reveal information hidden in the submovement profile. The details of the MSE algorithm have been reported elsewhere (Appendix S1)[28], [29]. Briefly, the MSE algorithm quantifies the sample entropy (SampEn) of the coarse-grained time series of kinematic data, such as to gain SampEn across different time scales (1–25 in this study), or the SampEn curve. The area under the SampEn curve in time scales 1–10 was empirically determined as the MSE area in low time scales. Each time scale for MSE in this study was 10 ms, conforming to the sampling rate of 100 Hz. The MSE area is a robust measure of biological complexity. A higher MSE area in submovement data indicates a noisier structure with more information contents in tuning of the movement trajectory. The standardized change in submovement complexity was the difference in MSE area between pre-test and post-test sessions divided by the MSE area of the pre-test session.

#### EMG analysis

The EMG data of the FDS and ED muscles of the pre-test and post-test conditions were rectified for spectral analysis. Rectification of surface EMG is believed to enhance the spectral peaks that represent common oscillatory inputs or the mean firing rate of an active muscle [30], [31], [32]. The power spectra of the rectified EMG signals were computed using Welch's method. A Hanning window with a window length of 1.6 seconds and an overlap of 0.4 seconds was used. Spectral resolution was 0.244 Hz. The spectral profiles of rectified EMG of the three trials in the pre-test and post-test sessions were averaged and then standardized with the mean spectral amplitude to reduce inter-subject variability. We obtained mean spectral peaks in the alpha (8–12 Hz), beta (13–20 Hz), and gamma (35–50 Hz) bands from standardized EMG spectral profiles of the three tracking trials in the pre-test and post-test sessions. Signal analyses were completed with custom-written programs using Matlab R2007 (Mathworks Inc., Natick, MA, USA).

### Statistical Analyses

For the simple, complex, and random conditions, the Wilcoxon signed-rank test was used to examine the practice effect by comparing the tracking congruency, submovement variables (relative submovement power, R_PM/S_, and MSE area), and EMG variables (peak oscillations in alpha, beta, and gamma bands) between the pre-test and post-test for each practice protocols. The Kruskal-Wallis test and a post-hoc test with Bonferroni correction were used to contrast standardized changes in tracking congruency, submovement variables among the simple, complex, and random conditions. Pearson's correlation analysis was used to examine the contributions of standardized changes in the submovement variables (the size and the complexity) to the standardized change in tracking congruency (learning benefits). All statistical analyses were completed using the SPSS 15.0 statistical package (SPSS Inc., USA) with the significance level set at *P* = 0.05.

## Results

### Tracking Congruency

**Figure 3:**
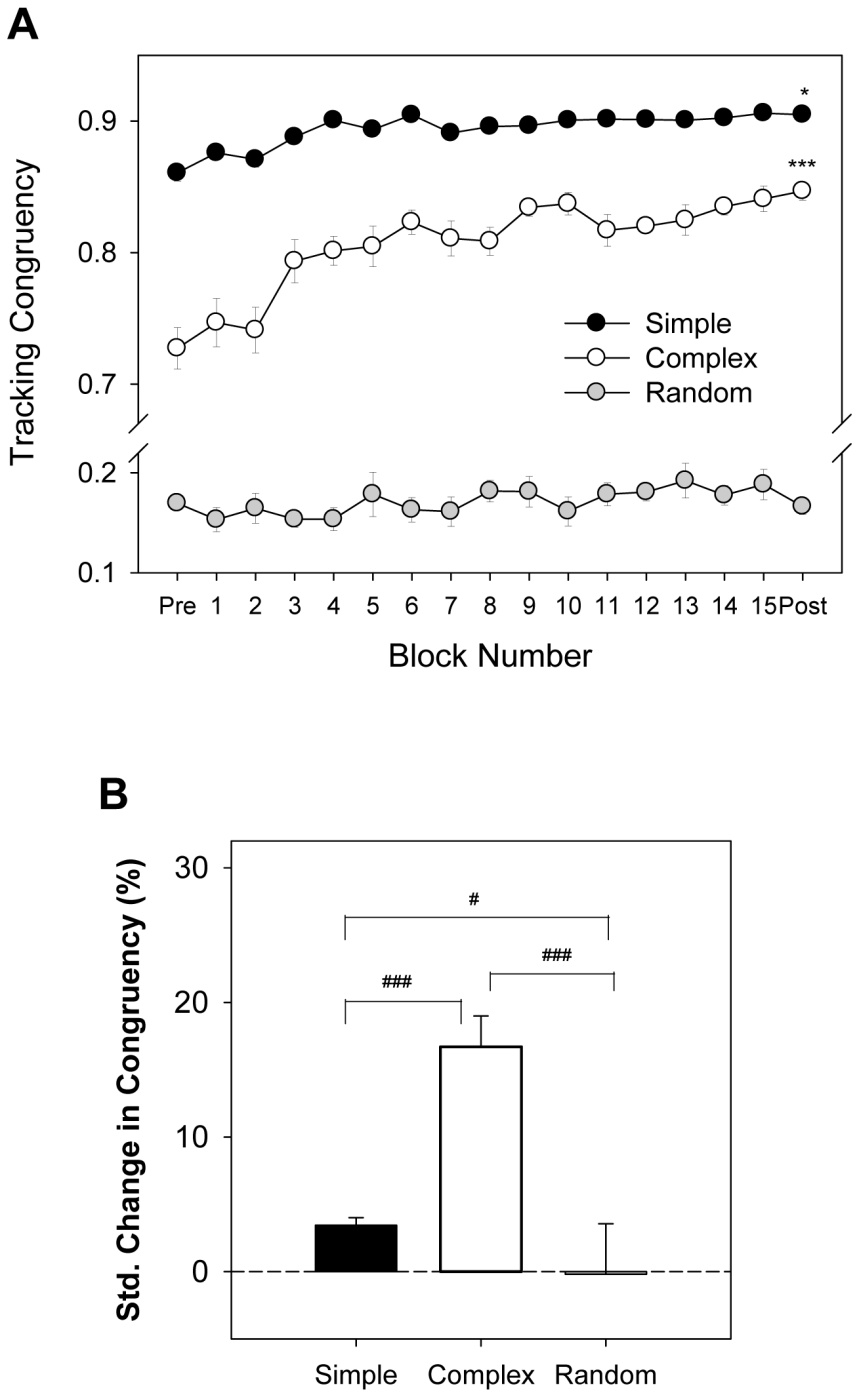
Contrast of mean evolutional changes in tracking performance among three practice protocols. (A) Tracking congruency, (B) Standardized change in tracking congruency due to practice effect among three practice protocols. (^*^: post-test > pre-test, p<0.05; ^***^: post-test > pre-test, p<0.001)(^#^: significant difference between protocols, p<0.0167; ^###^: significant difference between protocols, p<0.001)

In terms of CM, Figure 3A contrasts the evolutional changes in tracking congruency among the three different practice protocols. The tracking congruency of the simple and complex protocols increased progressively with the number of practice blocks, and post-test trials exhibited a greater CM than pre-test trials (Simple: Z = 3.052, p = 0.002; Complex: Z = 3.052, p = 0.002). For practicing under the random condition, tracking congruency did not vary between the pre-test and post-test sessions (Z = .001, p>0.05). The results of the Kruskal-Wallis test on the standardized change in CM suggested a task-dependent benefit of practice protocols (χ^2^(2) = 14.69, p = 0.001), with the greatest improvement in tracking congruency for the complex protocol (Complex > Simple > Random)(p<0.0167)(Fig. 3B).

### Submovement Characteristics

**Figure 4:**
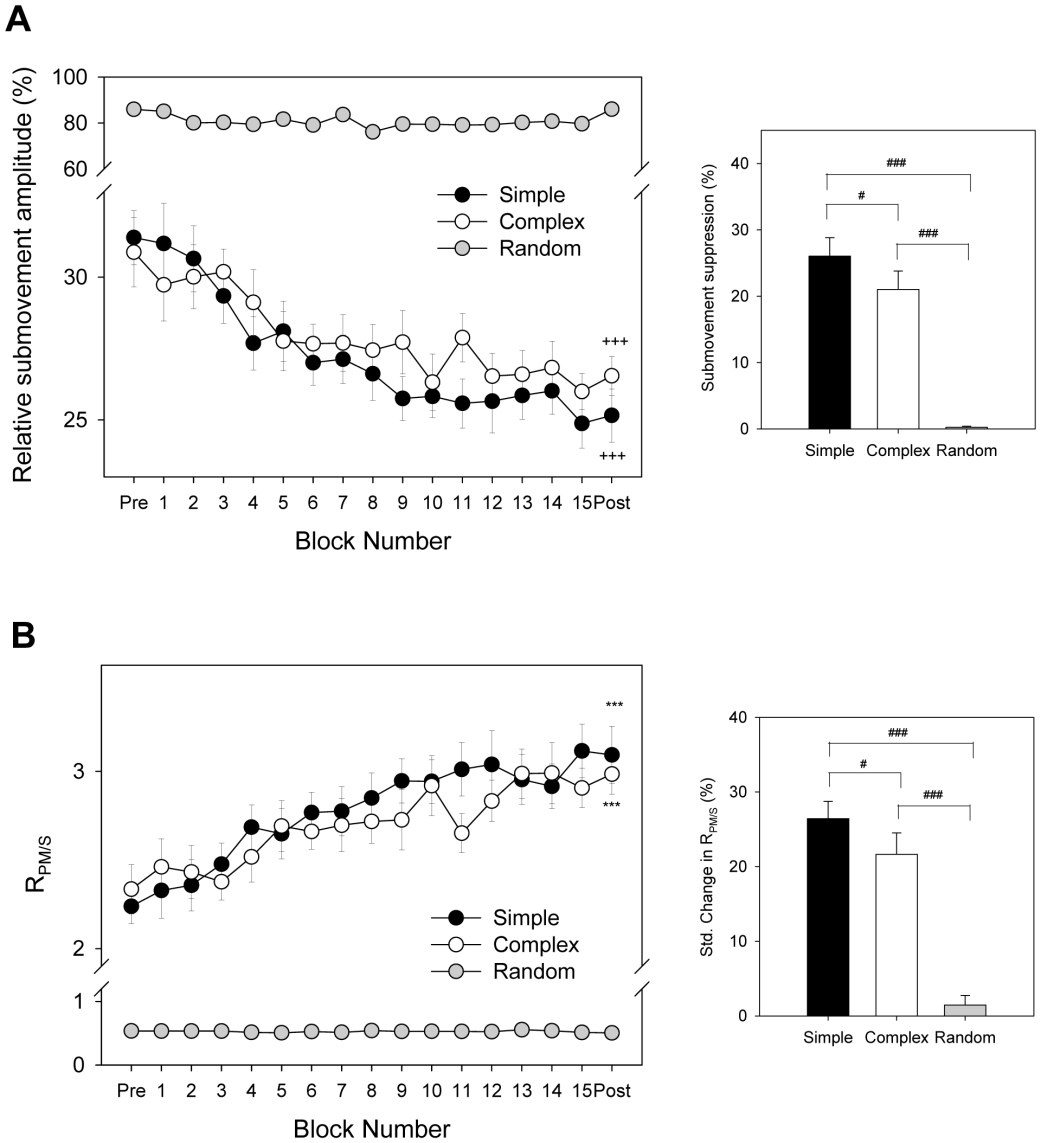
Contrast of mean evolutional and standardized changes in submovement amplitude among three practice protocols. (A) Relative submovement amplitude in percentage, (B) Amplitude ratio of primary movement to submovement (R_PM/S_) (^+++^: pre-test > post-test, p<0.05; ^***^: post-test > pre-test, p<.001)(^#^: significant difference between protocols, p<0.0167; ^###^: significant difference between protocols, p<0.001).

Figures 4A and 4B contrast the evolutional changes in the percentage of the submovement amplitude and amplitude ratio of primary movement relative to submovement (R_PM/S_) among the different practice protocols. For both the simple and complex protocols, practice led to decreases and increases in submovement amplitude and R_PM/S_, respectively (Figs. 4 A and B, left). The percentages of submovement amplitude in the post-test session were smaller than those in the pre-test session (Simple: Z = −3.061, p = 0.002; Complex: Z = −3.061, p = 0.002). The R_PM/S_ values were conversely larger in the post-test session than those in the pre-test session (Simple: Z = −3.059, p = 0.002; Complex: Z = −2.903, p = 0.004). For the random protocol, however, the percentage of submovement amplitude (Z = −1.020, p = 0.308) and R_PM/S_ (Z = −1.020, p = 0.308) were invariant to the practice effect. The results of the Kruskal-Wallis test revealed that standardized change in the submovement amplitude (χ^2^(2) = 20.126, p<0.001) and R_PM/S_ (χ^2^(2) = 17.479, p<0.001) varied with the practice protocol. The simple protocol resulted in the greatest reduction in submovement amplitude and enhancement in R_PM/S_ (p<0.0167)(Figs. 4A and 4B, right).

**Figure 5:**
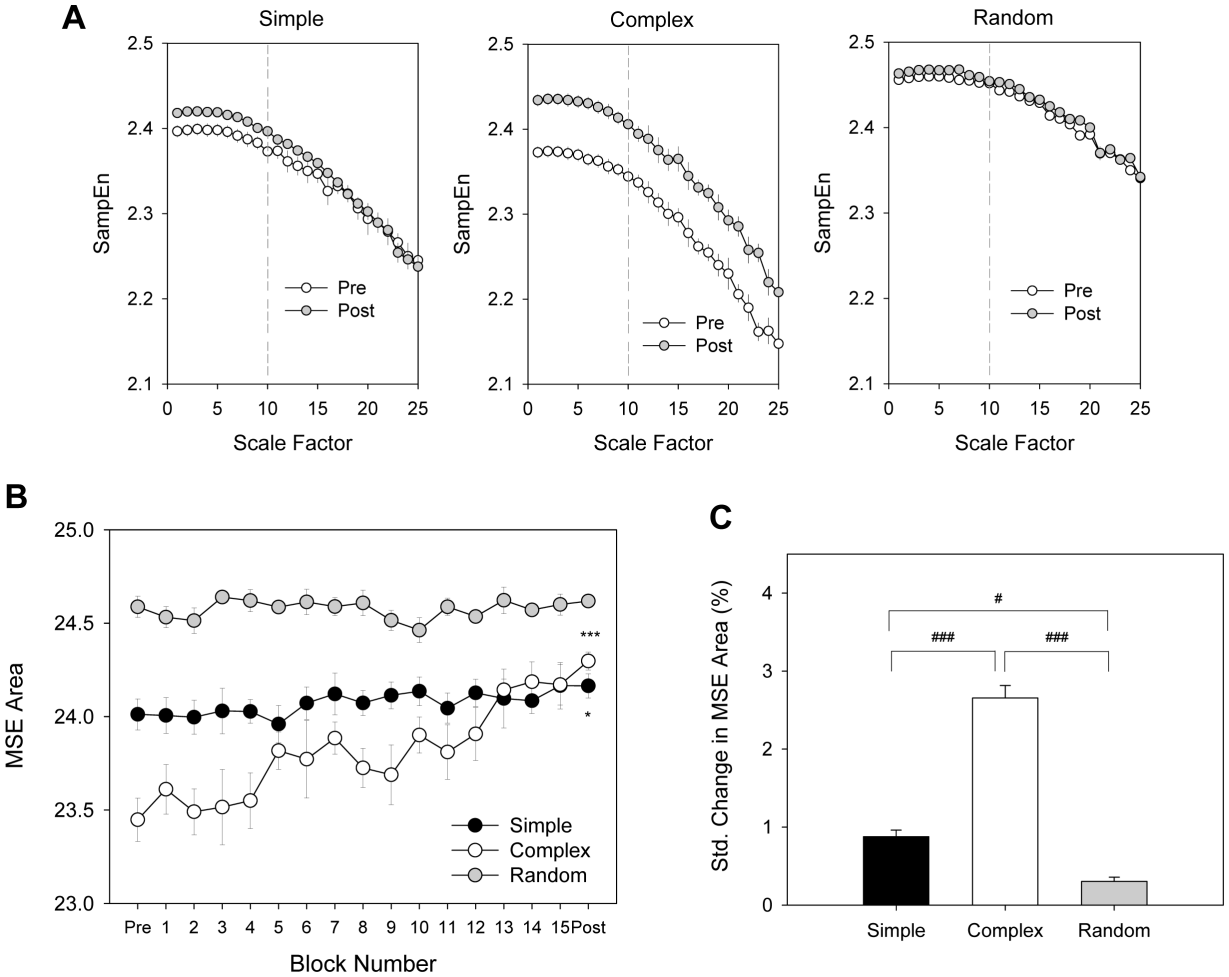
Multi-scale entropy (MSE) analysis of submovement profile for different practice protocols. (A) Pooled sample entropy versus scale factors of 1–25, (B) Evolutional change in MSE area under scale factors 1–10, and (C) Standardized change in MSE area due to practice effect. (^*^: post-test > pre-test, p<0.05; ^***^: post-test > pre-test, p<0.001)(^#^: significant difference between protocols, p<0.0167; ^###^: significant difference between protocols, p<0.001).

Figure 5A contrasts the SampEn curves by the time scale factors of the three practice protocols. Of note is that the SampEn curves, especially for the shorter scale factors (1–10), increased after practice under the simple and complex conditions. Figure 5B shows evolutional change in the MSE area under scale factors 1–10 for different practice protocols. The practice effect on the MSE area (or submovement complexity) was evident for the simple and complex protocols (Simple: Z = −2.040, p = 0.041; Complex: Z = −3.059, p = 0.002), but not for the random protocol (Z = .000, p = 1.0). The Kruskal-Wallis test suggested a protocol-dependent change in submovement complexity (χ^2^(2) = 12.12, p = 0.002), and complex protocol produced the greatest practice potentiation in submovement complexity (p<0.001)(Fig. 5C).

As only the simple and complex groups exhibited significant learning benefits, Pearson's correlations were calculated between the standardized changes in tracking congruency (Δ tracking congruency) and standardized changes in submovement variables, based on the subjects in the two groups (n = 24). Δ tracking congruency was significantly related to standardized changes in submovement complexity (MSE area)(r = .813, p = 0.000) but not in submovement size (r = −.188, p = 0.380). Namely, the standardized change in submovement complexity was a potent predictor for practice-related improvement in tracking congruency.

### Emg Time Series

**Figure 6:**
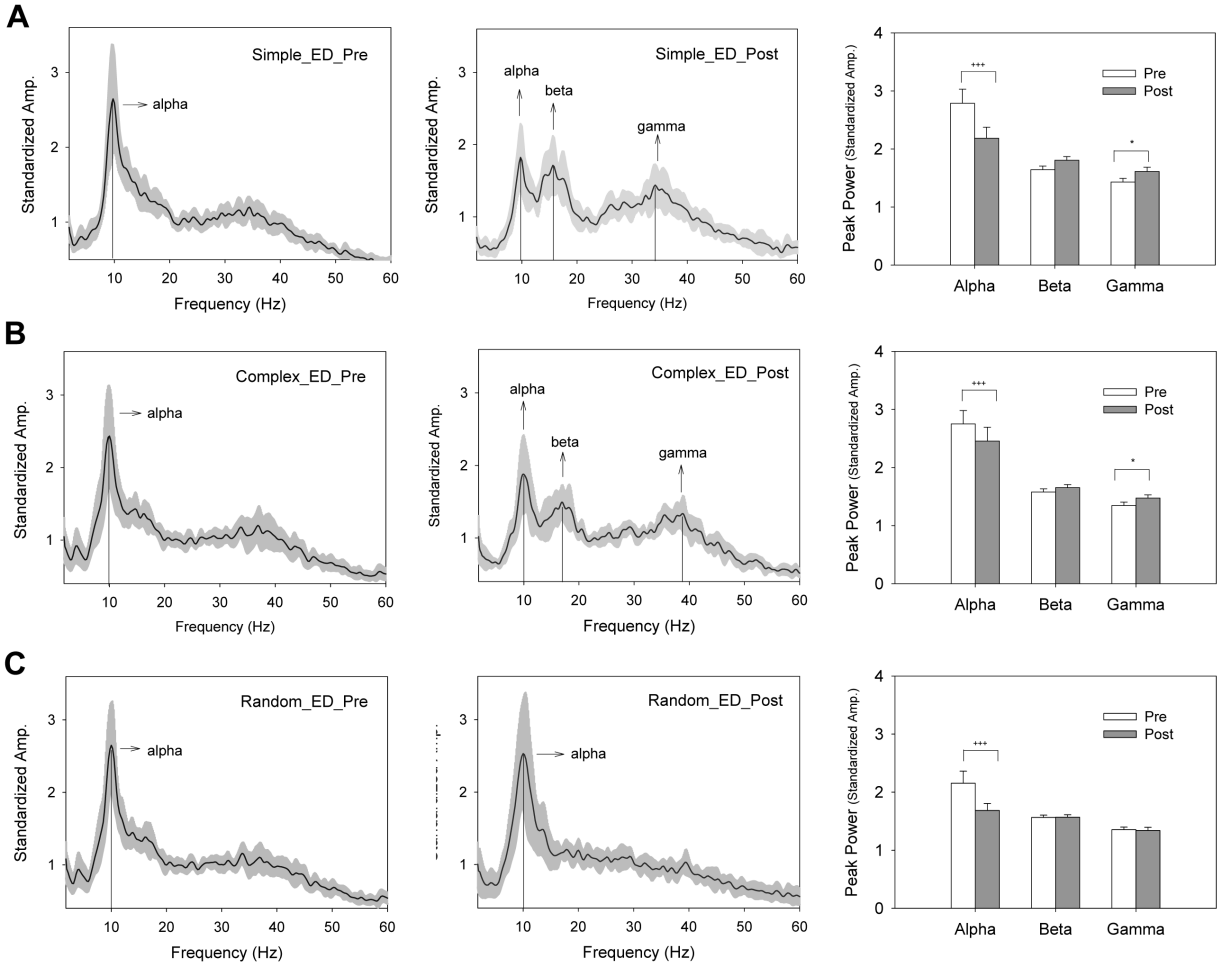
Pooled power spectra of rectified EMG from the ED muscle before and after practice. (A) Simple protocol, (B) Complex protocol, and (C) Random protocol. Visible changes in higher frequency muscular rhythmicity at 13–20 Hz and 35–50 Hz were noted in the simple and complex conditions. The shaded area represents standard deviation of the pooled spectral profile. (^*^: post-test > pre-test, p<0.05; ^+++^: pre-test > post-test, p<0.001)(ED: the extensor digitorum).

**Figure 7:**
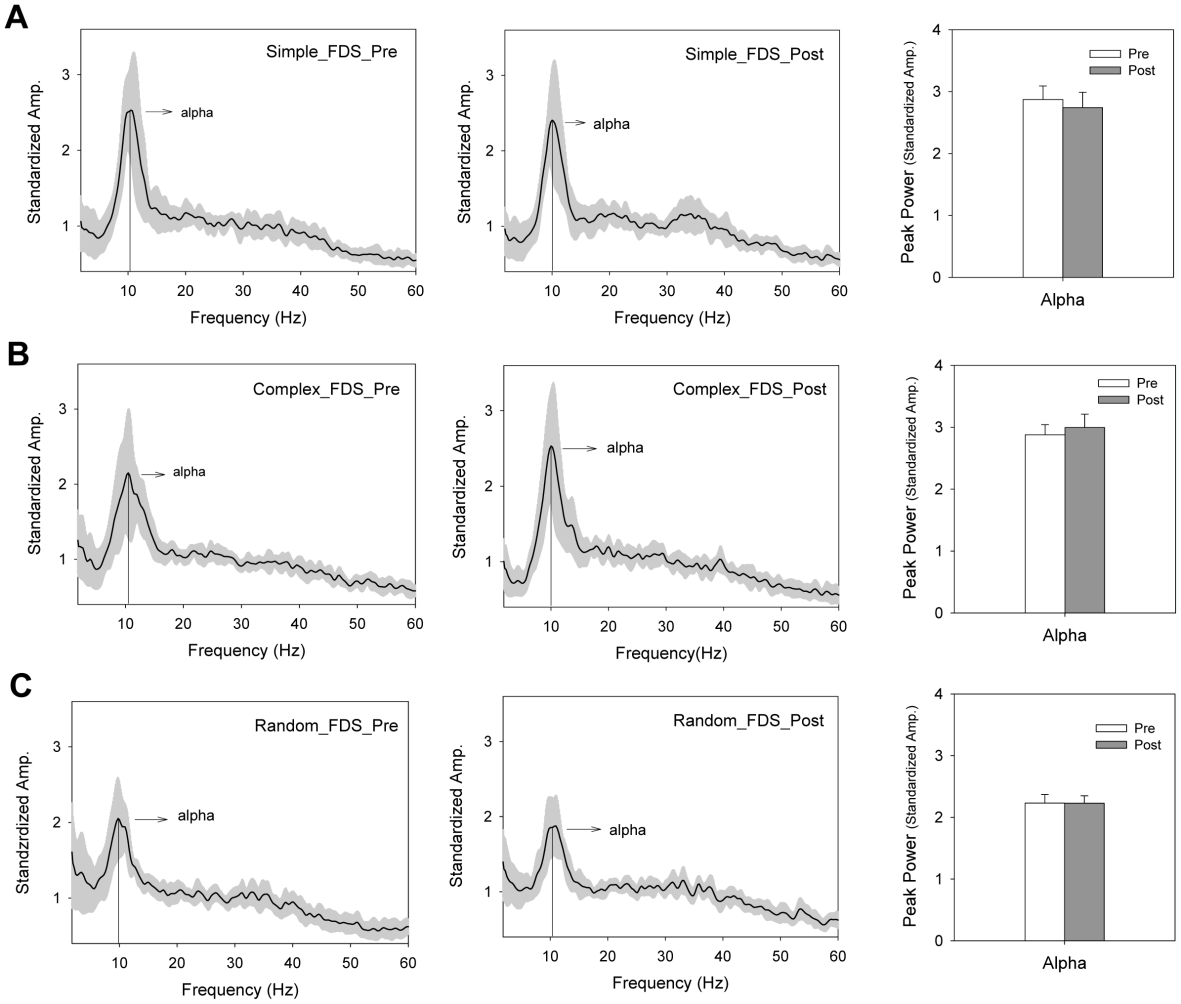
Pooled power spectra of rectified EMGs from the FDS muscle before and after practice. (A) Simple protocol, (B) Complex protocol, and (C) Random protocol. There was a prominent 8–12 Hz oscillation peak in the pre-test and post-test sessions. The shaded area represents the standard deviation of the pooled spectral profile. (FDS: the flexor digitorum superficialis).

Figure 6 contrasts the pooled power spectra of the rectified EMG of the ED muscle between the pre-test and post-test sessions under different target conditions. There were several visible changes in the spectral distribution of the ED EMG following practice under the simple and complex conditions (Figs. 6A and 6B). The results of the Wilcoxon signed-rank test suggested that the standardized amplitude of alpha spectral peak (8–12 Hz) was smaller in the post-test condition than in the pre-test conditions (Simple: Z = −2.432, p = 0.015; Complex: Z = −2.040, p = 0.04). In contrast, the standardized amplitude of gamma oscillation (35–50 Hz) was enhanced following practice, with a greater gamma spectral peak in the post-test condition (Simple: Z = −2.746, p = 0.006; Complex: Z = −2.903, p = 0.004). The beta spectral peak (13–20 Hz) did not significantly differ in the pre-test and post-test conditions (Simple: Z = −1.412, p = 0.158; Complex: Z = −1.225, p = 0.209), although the beta oscillation in the pooled spectral profile of the ED EMG appeared to be more prominent in the post-test condition. For the random condition, alpha oscillation was consistently present in the pre-test and post-test conditions (Fig. 6C). The result of the Wilcoxon signed-rank test suggested that alpha peak power was decreased in the post-test condition (Z = −3.059, p = 0.002). Figures 7A–7C contrast the pooled power spectra of the rectified EMG for the FDS muscle between the pre-test and post-test sessions under the different target conditions. For all the practice paradigms, only the alpha rhythm was present in the FDS muscle during tracking. The result of the Wilcoxon signed-rank test suggested no significant difference in alpha spectral peak between the pre-test and post-test conditions (Simple: Z = −1.255, p = 0.209; Complex: Z = −1.225, p = 0.209; Random: Z = 0.000, p = 1.0).

## Discussion

In light of tracking congruence, submovement dynamics, and muscular oscillation, the present results confirmed the effects of information-based practice on learning benefits and a trajectory-tuning strategy for visuomotor tracking. Practice in the simple and complex conditions resulted in positive learning benefits, concurrent with reduction of the submovement size, potentiation of the submovement complexity, and enhancement of the gamma muscular rhythmicity at 35–50 Hz in the working muscle. However, the tracking skill was not improved after practice with excessive target information, and the submovement size, submovement complexity, and gamma muscular oscillation were not altered in the random condition.

### Learning-based reduction in the size of submovements

For practicing under the simple and complex conditions, the reduction in the size of submovements could be best explained as a concomitant decrease in performance variability with improvement in skill. In addition to peripheral afferents, the central nervous system could scale speed pulses that are overlapped and superimposed onto a desired movement trajectory according to the accuracy constraints of visuomotor tracking [13], [17]. Under the framework of intermittent movement control [14], [15], submovements are corrective motor acts for fine-tuning a force trajectory, predominantly with feedback processes. Central to this interpretation, the learning-based reduction of the size of submovements indicates a smoother and less variable tracking trajectory in the post-test trials. Practice with an appropriate amount of target information could effectively minimize the negative impact of kinematic variability because the subjects could adopt kinematic patterns of the target with practice and prevent excessive corrective actions based on feedback processes.

**Figure 8:**
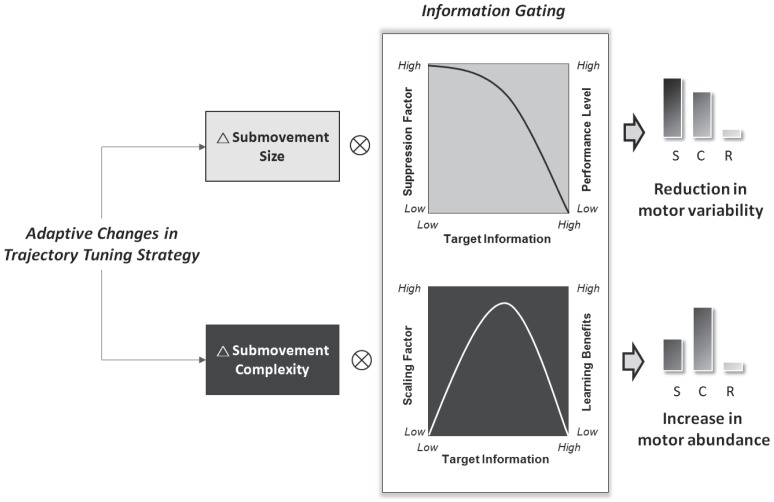
Summary illustration of learning-based channeling and modulation of submovement characteristics with respect to target information. The amounts of change in submovement size (△submovement size) and submovement complexity (△submovement complexity) are differently modulated with respect to target information. In accordance with the learning-information relationship, △submovement size is downward-modulated with target information increment (simple (S) > complex (C) > random (R)). Namely, submovement size is not suppressed under practice with excessive target information. On the other hand, the effect of target information on △submovement complexity during practice is an inverted-U function, with maximal change in complexity at medium target information.

Of particular importance for understanding the role of the size of submovements in performance is that the standardized change in the amount of submovement suppression (simple (S) > complex (C) > random (R)) was inversely related to the amount of target information (Fig. 4A). The fact that learning-based suppression of submovement size is gated by target information is nicely predicted by the learning-performance relationship [22] (Fig. 8). Once the amount of target information exceeds what the subjects could process, as occurred in practice under the random condition, submovement size (or motor variability) could not be tuned with practice according to task demands. Under the simple and complex conditions, however, the submovement size is gradually minimized with practice in consequence to less drastic trajectory tuning in the course of tracking. An increase in R_PM/S_ (Fig. 4B) signifies consolidation of movement execution with less uncertainty and relative enhancement of the internal drive to produce rhythmic tracking movements of the target rate [8], [20].

### Learning-based enhancement in the complexity of submovements

For concomitant learning benefits, the increase in submovement complexity following practice with the simple and complex protocols was a positive aspect of motor abundance. Hence, skill improvement is often associated with a lower likelihood of similar relative characteristics in the distance between submovement data points [5]. This fact suggests that the subjects were able to develop a richer trajectory-tuning pattern as a result of practicing with appropriate amounts of target information [6]. In fact, complexity is naturally inherent in skilled and mature motor behaviors. When sensory information cannot be fully exploited in the setting of visual or somatosensory impairments, motor behaviors could lose complexity. For example, older adults [33] and patients with neurological disorders [3], [34], [35] exhibit a larger but more regular postural sway during quiet stance because they lack sufficient adaptability to stance perturbation, known as the loss of adaptability hypothesis.

It is apparent that the submovement complexity and submovement size were differentially channeled and modulated following practice as the amount of target information varied. Changes in submovement complexity (△submovement complexity) in response to target information had an inverted-U function (complex (C) > simple (S) > random (R))(Figs. 5C and 8), akin to the relationship between potential learning benefits and task difficulty [22]. According to the challenge point hypothesis, potential learning benefits are optimal with practice at a moderate level of task difficulty, or the optimal challenge point. In line with the theoretical framework, our results also support that the greatest complexity increment in submovements after practice took place around the moderate target information level (moderate task difficulty). When the amount of target information exceeded the capacity of the visuomotor system to process it, no target information could be successfully used to enrich the state of movement complexity. Supplementary to the challenge point framework, the change in submovement complexity is noted to be a potent predictor for information-dependent learning benefits. We posit that the potential learning benefits after effective practice are determined by the transmission of information on target movement, such as remedying trajectory deviations with versatile submovement patterns due to strategic abundance.

### Adaptive changes in muscular oscillations following practice

In the simple and complex conditions, the improvements in the submovement complexity and tracking success were very likely to be inherited from the emergence of muscular oscillation at 35–50 Hz in the ED muscle (Figs. 6 A, 6B). Coincidently, the gamma band in rectified EEG-EMG coherence (EEG-EMG piper rhythm) is enhanced when motor tasks require more computational loads or cognitive resources during phasic contraction [36], [37], such as periodic isometric contraction [37] and repetitive isotonic contraction [36], [38], [39]. During the holding of a compliant object [40] or force exertion at a high level of precision [24], the EEG-EMG piper rhythm also increases, representing the intensive use of sensory cues. Although this study did not directly assess the EEG-EMG corticomuscular coherence, the muscular oscillation at the 35–50 Hz muscular oscillation was likely a peripheral part of the gamma corticomuscular coherence due to synchronous firing of spinal motoneurons in phase with the cortical rhythm [30]. Similar to the functioning of EEG-EMG corticomuscular coherence in the gamma band, the potentiation of gamma muscular oscillation in the simple and complex conditions was a physiological signature of practice-related alterations for strategic advancements. One possible explanation for the use of a fast coding scheme (or gamma muscular oscillation) is an increased capability of effectively integrating task-relevant sensory information after practice [25]. The gamma band of the muscular oscillation was not potentiated in the random condition, in parallel with insignificant changes in learning benefits (Fig 3B) and submovement dynamics (Fig. 6C),

Another practice-related modulation of muscular oscillation was noted in the alpha muscular oscillation (8–12 Hz), probably physiological tremor in the ED muscle [41], [42]. Although the alpha muscular oscillation was smaller in the post-test session for all practice protocols, the reduction in this rhythm was not functionally anchored to learning because suppression of the alpha muscular oscillation concurred with practice under the random condition. There were 4–5 subjects who presented evident beta muscular oscillation (13–20 Hz) that contributed to a visible change in the beta band of the pooled EMG spectral profile after practice under the simple and complex conditions (Figs. 6 A and B). Although the population mean of the beta peak power was not significantly different before and after practice, the presence of EMG beta rhythm in some of the subjects is still worth noting. This scenario favors inter-individual strategic differences in the control of phasic movements [43], [44]. Increases in the beta band of rectified EMG-EEG coherence have also been reported in some subjects who demonstrated better performance during sinusoidal force tracking [44].

In conclusion, this paper presents a novel finding, that the scaling properties of submovement and muscular oscillations reflect information-based motor learning. There are two separate processes for submovement scaling (size and complexity) during motor learning with different amounts of information. In agreement with the theoretical model of optimal movement variability, practice with a suitable amount of target information reduces the negative impact of behavioral variability, or the size of submovements. Submovement size is reduced because motor variability and fruitless error-correction are minimized, fitting the behavioral outputs consistently according to task constraints. In agreement with the challenge point hypothesis, the increase in submovement complexity after practice is the positive impact of behavioral variability, showing an inverted-U relationship with increases in target information. Submovement complexity is a behavioral repertoire of exploratory flexibility to achieve task goals. Contingent on the channeling and modulation of submovements, the potentiation of gamma muscular oscillation is a biological marker of information-based learning of trajectory control.

## Supporting Information

Appendix S1 (DOC)
