# Supplementary material for: Characterization of Information-Based Learning Benefits with Submovement Dynamics and Muscular Rhythmicity
Source: PLoS One. 2013 Dec 18;8(12):e82920. doi: 10.1371/journal.pone.0082920 (PMC3867443; doi:10.1371/journal.pone.0082920)
Supplement: Appendix S1 — (DOC) [file pone.0082920.s001.doc]

**Appendix S1**

The calculation of the multi-scale entropy area (MSE Area) consists of three steps [28,29].

The first step: Obtain the coarse-grained sequences of a down-sampled

submovement trace {*X^(τ^* )}

$X^{(j)}=\frac{1}{n}\sum_{i=(j-1)n}^{jn} z_{i}$ (a)

where {z_1_, z_2_, . . ., z_N_} is the time series of submovement and τ is the time scale

The second step: Calculation of SampEn for each coarse-grained sequence {*X^(τ^* )}

MSE^(τ )^ = {*SampEn*(*m, r, N*)^(τ )^,_τ=1,2,…25_} (b)

$SampEn(m,r,N)=\ln(\frac{\sum_{i=1}^{N-m} n_{i}^{m}}{\sum_{i=1}^{N-m-1} n_{i}^{m+1}})=\ln(\frac{n_{n}}{n_{d}})$ (c)

$n_{i}^{m}=d\left[f_{m}(i),f_{m}(j)\right]\leq r$ (d)

$d\left[f_{m}(i),f_{m}(j)\right]=\max\left\{\left|f(i+k)-f(j+k)\right|\right\},0\leq k\leq m-1$ (e)

SampEn measures the negative natural logarithm of an estimate of the conditional probability that epochs of length *m* that match point-wise within a tolerance level (*r*) also match at the next point. In equation (d), *d*[] denotes Euclidean distance. Here, *r* = 20% of the standard deviations of *X^(τ^* ), and *m* = 2. [28,29]

The third step: Summation of each MSE^(τ )^ across different time scales (τ) to

obtain MSE area

$MSE Area=\sum_{\tau=1}^{10} MSE^{(\tau)}$ (f)
